# Contrasting benthic ecological quality within tropical estuaries in Southeastern Brazil based on biotic indices

**DOI:** 10.7717/peerj.21883

**Published:** 2026-10-22

**Authors:** Anderson Antonio Batista, Heliatrice Louise Hadlich, Angelo F. Bernardino

**Affiliations:** Department of Oceanography, Universidade Federal do Espírito Santo, Vitória, Espirito Santo, Brazil

**Keywords:** Benthos, Macrofauna, Southwestern Atlantic, Estuaries, Ecological index, Pollution

## Abstract

**Background:**

Tropical estuaries are exposed to multiple sources of impact within a background of strong natural environmental gradients. Monitoring and managing these ecosystems is challenging because gradients in salinity and hydrodynamics overlap with intense, spatially localized anthropogenic pressures. Benthic biotic indices provide standardized tools for assessing the ecological health of estuaries and are sensitive to multiple impact sources, but as these indices are based on regional taxa assignments, they need testing to verify their capacity to indicate soft-bottom assemblages under stress. Although these indices have been applied across a broad latitudinal range, their performance remain less established in the tropics.

**Methods:**

We quantified macroinfaunal abundance, richness, diversity and assemblage composition across 64 stations in two tropical estuaries of southeastern Brazil, calculated the AZTI Marine Biotic Index (AMBI) and Multivariate AZTI Marine Biotic Index (M-AMBI), and evaluated their associations with salinity, sediment grain size, organic-matter quality, fecal sterols and trace and major elements. We then quantified multivariate associations between macrofaunal assemblages, benthic ecological indices and environmental gradients.

**Results:**

Benthic ecological status was spatially heterogeneous, with localized Poor/Bad and High/Good indices within estuarine sectors. Community composition was associated with salinity, sediment grain size, organic-matter quality and fecal sterols. Multivariate tests identified significant associations between community variation and salinity, biopolymeric carbon, sterols, total organic carbon, total nitrogen, fine sediments and selected metals. Overall, the indices captured pronounced station-level variation in both urban and less-developed estuaries, and reveal their value to identify stress within tropical estuaries of Brazil.

## Introduction

Estuaries are among the most productive and highly socially valuable coastal ecosystems, supporting high biodiversity, fisheries biomass, nutrient and pollutant removal and cultural benefits (Kennish, 2002; Barbier et al., 2011; Otieno et al., 2026). However, many estuaries worldwide are heavily altered by urbanization, leading to widespread impacts related to land use change, urban pollution and waste discharge. In many cases, the extent and intensity of these impacts are spatially heterogeneous and site-specific, depending on the nature of local acute impacts such as sewage discharge, port activities, and habitat conversion (Lotze et al., 2006). Determining the spatial scales at which ecosystem impacts occur is an important step toward more effective estuarine management, as benthic indicators may vary from station to regional scales and lead to different management interpretations (Quintino, Elliott & Rodrigues, 2006).

In addition to anthropogenic impacts, estuaries hold strong natural gradients in salinity, sediment texture, and water flow that create spatially complex mosaics in benthic habitats. As a result, detecting anthropogenic change against this naturally variable background remains a central challenge for estuarine monitoring and management (Dauvin, 2007; Elliott & Quintino, 2007; Borja & Dauer, 2008). Soft-bottom macrofaunal assemblages are widely used to infer estuarine ecological health, because these organisms integrate short-term environmental conditions over their life cycles. Most benthic invertebrates also have limited mobility, remain closely associated with sediments where contaminants and organic matter accumulate, and differ markedly in sensitivity, feeding mode, life history and tolerance to oxygen depletion or toxic substances (Dauer, 1993). These benthic organisms also regulate ecosystem processes through bioturbation and trophic transfer, thereby helping to quantify ecosystem functioning across these spatial patterns of change (Borja & Dauer, 2008; Barcellos et al., 2021).

The conceptual foundation of many benthic indicators lies in the well-documented succession of soft-bottom macrofaunal communities under stress (Pearson & Rosenberg, 1978). Under severe enrichment, oxygen consumption and reduced sediment conditions can lead to hypoxia, defaunation and altered food webs (Gray, Wu & Or, 2002; Diaz & Rosenberg, 2008). The AZTI Marine Biotic Index (AMBI) was developed to translate macrofaunal composition into an interpretable disturbance gradient by assigning taxa to five ecological groups ranging from sensitive species to first-order opportunists (Borja, Franco & Pérez, 2000). Its application to multiple impact sources in estuarine ecosystems demonstrated consistent changes in soft-bottom assemblages under stress (Borja, Muxika & Franco, 2003; Borja & Muxika, 2005). M-AMBI (Multivariate AZTI Marine Biotic Index) subsequently combined AMBI with taxonomic richness and Shannon diversity, producing an ecological-quality ratio intended to improve discrimination among ecological classes and support comparisons with reference conditions (Muxika, Borja & Bald, 2007; Borja & Dauer, 2008). AMBI and M-AMBI have since been applied across a broad range of latitudes, salinity regimes and habitat types, including subtropical and tropical estuaries, and have become among the most widely used benthic assessment tools worldwide (Borja & Tunberg, 2011; Borja, Dauer & Grémare, 2012; Borja, Chust & Muxika, 2019).

Despite their broad adoption, benthic indices are not context-free. Ecological-group assignments may be uncertain for poorly studied taxa, index performance declines when many organisms remain unassigned, and M-AMBI classifications depend on the selection of appropriate high- and bad-status reference conditions (Borja & Muxika, 2005; Muxika, Borja & Bald, 2007; Borja, Dauer & Grémare, 2012). In naturally stressed transitional waters, low diversity and dominance by tolerant species may reflect salinity variability, muddy sediments or periodic oxygen limitation rather than anthropogenic degradation. This difficulty, known as the estuarine quality paradox, can generate false-positive diagnoses of disturbance when indices developed primarily for coastal marine systems are applied without local ecological interpretation (Dauvin, 2007; Elliott & Quintino, 2007). Comparisons among indices have also shown that different metrics may classify the same station differently because they emphasize distinct attributes of assemblage structure (Borja & Dauer, 2008; Brauko et al., 2015; Lu et al., 2021). For these reasons, index scores should be interpreted together with taxon-level composition, environmental gradients and evidence of specific contamination sources.

Application of AMBI-based approaches in tropical regions has expanded substantially, but tropical estuaries remain underrepresented relative to European and temperate systems. Early testing in the South Atlantic demonstrated the applicability of AMBI to South American soft-bottom benthic assemblages, while also emphasizing the need for regional taxonomic knowledge and locally appropriate ecological-group assignments (Muniz et al., 2005; Valença & Santos, 2012; Brauko et al., 2015; Checon et al., 2018; Souza et al., 2021). Studies from tropical and subtropical Asia similarly report that AMBI and M-AMBI can detect anthropogenic gradients, but their relative performance varies with season, habitat, taxonomic resolution, unassigned taxa and the environmental pressures considered (Li et al., 2021; Lu et al., 2021; Song et al., 2023). Collectively, this literature supports the use of AMBI and M-AMBI in tropical systems while reinforcing the need for integrated, site-specific validation.

Brazilian estuaries encompass extensive climatic, geomorphological and salinity gradients, yet there are large gaps and uneven efforts in benthic assessments across the country (Lana & Bernardino, 2018). The Eastern Brazil Marine Ecoregion is particularly relevant because it contains highly urbanized estuaries, mangrove-dominated systems and coastal habitats influenced by strong climatic variability (Mazzuco & Bernardino, 2022). Urban and pristine estuaries exhibit pronounced spatial variation in soft-bottom benthic assemblages associated with organic pollution, salinity gradients and seasonality (Hadlich et al., 2018; Bissoli & Bernardino, 2018). These systems therefore provide a valuable framework for testing benthic indices under contrasting levels of organic pollution and natural salinity gradients.

In this study, we evaluated the macrofaunal community structure and benthic ecological status across two contrasting estuaries of the Eastern Brazil Marine Ecoregion. We quantified spatial assemblage patterns with AMBI and M-AMBI indices and examined their relationships with salinity, sediment grain size, organic-matter quality, sedimentary fecal and total sterols, and trace and major elements. Our objectives were to: (*i*) characterize spatial variation in macrofaunal abundance, richness, diversity and taxonomic dominance across estuaries; (*ii*) compare the spatial differences in ecological status based on AMBI and M-AMBI ecological-status categories; and (*iii*) identify and compare environmental gradients associated with these ecological indices between urbanized and pristine estuaries. We expected ecological quality to vary spatially (intra-estuary) following gradients of disturbance rather than decline uniformly within entire estuarine sectors. By integrating biotic indices, assemblage structure and sediment geochemistry, this study advances the evaluation of the strengths and limitations of AMBI-based assessment in tropical estuaries.

## Materials & Methods

### Study area and sampling

This study focused on infaunal benthic macroinvertebrate diversity and ecological status, integrating macrofaunal assemblage descriptors with AMBI/M-AMBI and estuarine environmental variables. Sampling was conducted in two estuaries within the Eastern Brazil Marine Ecoregion: Vitória Bay (20°18′S, 40°20′W) and the Piraquê-Açu–Mirim estuarine system (19°58′S, 40°00′W) (Fig. 1). Vitória Bay (VB) is a metropolitan estuary affected by domestic and industrial effluents delivered through tributaries and urbanized margins (Varzim et al., 2019). The Piraquê-Açu–Piraquê-Mirim (PAM) estuary contains extensive mangrove forests and tidal flats and is subject to substantially lower coastal development (Bernardino et al., 2018a; Bernardino et al., 2018b; Bissoli & Bernardino, 2018; Bernardino et al., 2020).

**Figure 1:**
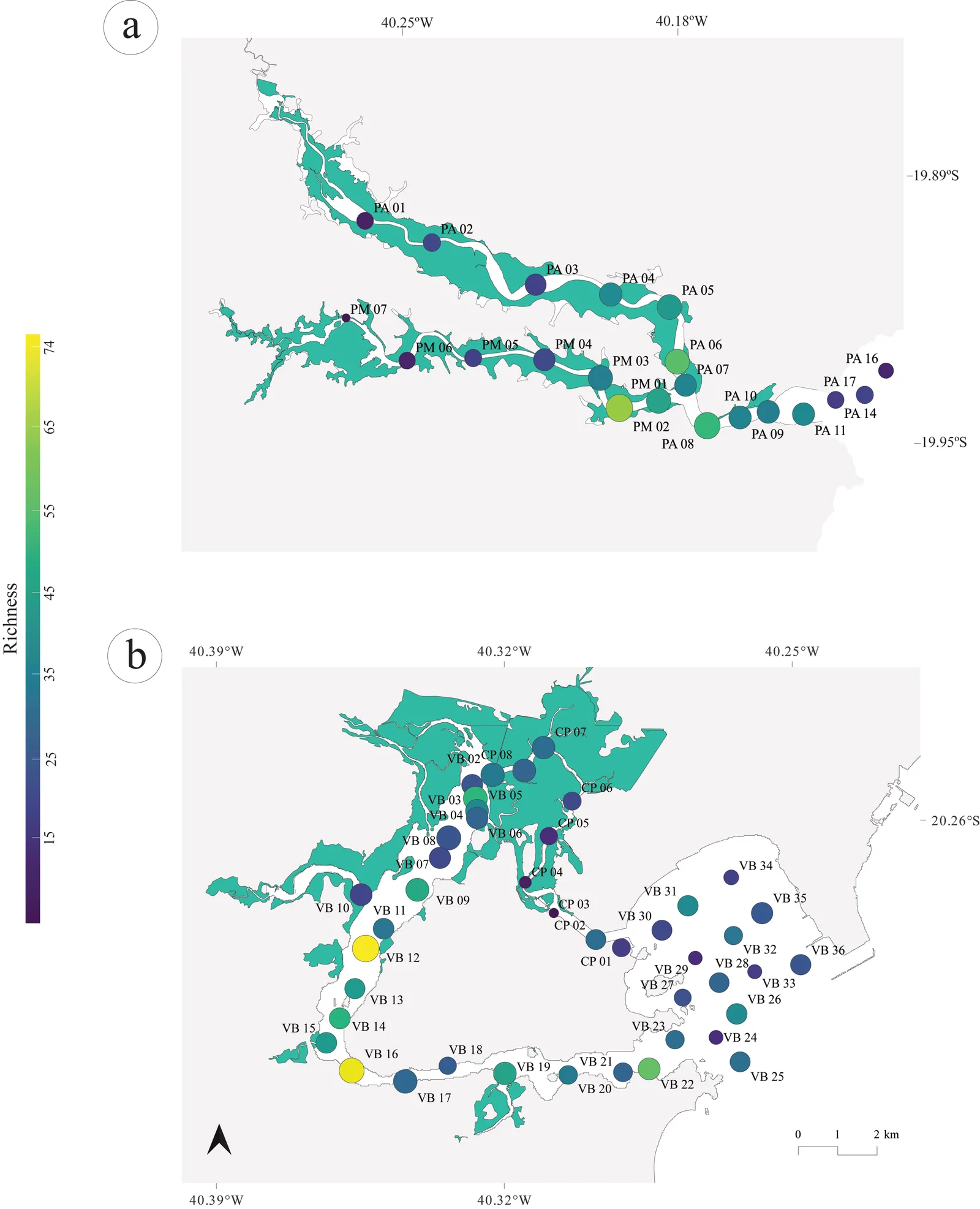
Map of sampled sites. Study area with locations of sampled stations in the two tropical estuaries. (A) Piraquê-Açu (PA, North) and Piraquê-Mirim (PM, South) estuaries. (B) Vitória Bay, showing stations distributed across the inner bay, harbor channel and outer bay, including Canal da Passagem stations (CP). Color code represents macrofaunal taxonomic richness at each sampling station. Turquoise areas represent mangrove/fringing estuarine vegetation. Map component sources: GEOIEMA and ggplot2 package in R.

Invertebrate sampling authorization was issued by ICMBIo (Instituto Chico Mendes de Conservação da Biodiversidade, Brazilian Ministry of Science) licence #24700-1. Both estuaries were sampled in November 2014 using a spatially distributed random design across the main estuarine channel of both systems. Therefore, our results represent spatial patterns at the time of sampling without considering potential seasonal or interannual variability over these assemblages. The complete macrofaunal dataset included 64 stations from the two estuaries: 35 stations in the main Vitória Bay sector (VB), eight stations in the Canal da Passagem (CP), 14 stations in Piraquê-Açu (PA) and seven stations in Piraquê-Mirim (PM; Fig. 1). In Vitória Bay, stations VB02 to VB15 were located in the inner estuary, VB17 to VB21 along the Vitória harbor channel and VB24 to VB36 in the outer Espírito Santo Bay. CP stations were sampled along the secondary Canal da Passagem, which connects the inner estuary to Espírito Santo Bay. In the Piraquê-Açu (PA) and Piraquê-Mirim (PM) estuaries, stations were distributed along the estuary-to-mouth gradient (Fig. 1). Sampling for sediment organic geochemistry was conducted at 30 stations due to limited funds for analysis (19 in VB, 11 in PA and PM estuaries).

Salinity was measured at the time of sampling with a calibrated SonTek CastAway CTD. At each station included in the sediment chemistry and particle-size analyses, two independent 0.1-m^2^ Day-grab collections were used as field subsamples. The upper three cm of sediment from the two grabs were physically combined before grain size and geochemical laboratory analysis to create a single station-level composite sample. For organic compounds, samples were stored in pre-cleaned aluminum containers, whereas surface sediments for trace-metal analysis were stored in pre-cleaned low-density polyethylene (LDPE) containers. Samples remained frozen (−20 °C) until laboratory procedures.

### Laboratory analysis

For macrofaunal sampling, two independent 0.1-m^2^ Day-grab samples were collected per station and kept separate through washing, fixation, sorting, identification and counting. The material was gently washed through a 0.5-mm mesh on board, fixed in 4% buffered formalin and subsequently transferred to 70% ethanol in the laboratory. Organisms were sorted under a stereomicroscope, counted and identified to the lowest practicable taxonomic level, generally genus or species, using specialized taxonomic literature (Amaral, Rizzo & Arruda, 2006). The taxonomic records used in the analyses are provided in the Supplementary material (Table S1) and are deposited in the Brazilian Online Biodiversity Repository (Bernardino & Batista, 2026).

Sediments were dry-weighed and fractionated through 2.0-, 1.0-, 0.5-, 0.25-, 0.125- and 0.063-mm sieves using a mechanical shaker. Fine sediment content (% mud) was calculated as the combined silt and clay fraction (<63 μm). Total organic carbon (TOC) was measured with a LECO TruSpec CNS elemental analyser. Proteins were extracted with 0.5 mol L^−1^ NaOH for 4 h and quantified following Hartree (1972), as modified by Rice (1982). Carbohydrates were determined following Gerchacov & Hatcher (1972), and lipids were extracted by ultrasonication in chloroform:methanol (2:1, v/v) and quantified following Marsh & Weinstein (1966). Protein, carbohydrate and lipid concentrations were expressed as bovine serum albumin, glucose and tripalmitin equivalents, respectively. Concentrations were converted to carbon equivalents using factors of 0.49, 0.40 and 0.75 and summed as biopolymeric carbon (BPC), an estimate of the labile organic-matter fraction (Fabiano & Danovaro, 1994; Neto, Bernardino & Netto, 2021). BPC was used as an indicator of sedimentary trophic conditions, with the original categories retained for descriptive interpretation: oligotrophic (<1 mg C g^−1^), mesotrophic (1–3 mg C g^−1^), eutrophic (3–5 mg C g^−1^) and hypertrophic (>5 mg C g^−1^) (Pusceddu et al., 2007). Protein:carbohydrate and carbohydrate:lipid ratios were also available as descriptors of biochemical degradation (Galois et al., 2000).

Sterols were extracted from sediments by Soxhlet extraction for 8 h with n-hexane:dichloromethane (1:1, v/v) after addition of 5α-androstanol as a surrogate standard, following Wisnieski, Ceschim & Martins (2016). Extracts were concentrated, purified and fractionated by silica/alumina liquid chromatography, derivatized with BSTFA/TMCS (99:1) for 90 min at 70 °C and spiked with 5α-cholestane before instrumental analysis. Sterols were quantified by gas chromatography with flame-ionization detection. Fecal sterols, total sterols and diagnostic ratios were used as indicators of sewage-derived organic contamination following Hadlich et al. (2018).

Sediment extracts were prepared with 1.0 mol L^−1^ HCl. Trace metals (Zn, V, Cr, Co, Cu and Pb) and major elements (Fe, Al and Mn) were measured by inductively coupled plasma optical emission spectrometry. Triplicate extractions, procedural blanks and certified reference material (PACS-2, National Research Council of Canada) were used for quality control.

### Data analysis

Taxonomic richness (S), Shannon diversity (H′, log_2_) and Pielou evenness (J′) were calculated from the macrofaunal dataset (Shannon & Weaver, 1949; Pielou, 1966). Ecological analyses were based on the combined abundance, richness and diversity from the two grabs standardized by the combined sampled area (0.2 m^2^ per station). This was justified as we had only one sample per station for geochemical analysis, and we were not evaluating spatial differences within stations.

Ecological quality was evaluated with the AZTI Marine Biotic Index (AMBI, version 5.0) and multivariate AMBI (M-AMBI). AMBI assigns taxa to five ecological groups according to their sensitivity to organic enrichment and disturbance: Group I, sensitive taxa; Group II, indifferent taxa; Group III, tolerant taxa; Group IV, second-order opportunists; and Group V, first-order opportunists (Borja, Franco & Pérez, 2000; Borja & Muxika, 2005). AMBI was calculated as [(0 × %GI) + (1.5 ×%GII) + (3 ×%GIII) + (4.5 ×%GIV) + (6 × %GV)]/100; higher values indicate increasing dominance by disturbance-tolerant and opportunistic taxa. From the total taxa list (168), 39 were not assigned to AMBI ecological-groups and were therefore not considered in calculations (Table S1). We have not made any regional adjustments in our taxa list and used only taxa available in AMBI version 5.0.

M-AMBI integrates AMBI, taxonomic richness and Shannon diversity into an ecological-quality ratio from 0 (bad) to 1 (high) (Table S2; Muxika, Borja & Bald, 2007; Borja & Dauer, 2008). The status thresholds represented in the worksheet were High (M-AMBI ≥ 0.77), Good (0.53–<0.77), Moderate (0.38–<0.53), Poor (0.20–<0.38) and Bad (<0.20). AMBI disturbance classes included Undisturbed (≤1.2), Slightly disturbed (>1.2–3.3), Moderately disturbed (>3.3–5.0), Heavily disturbed (>5.0–6.0) and Extremely disturbed (>6.0). Within the standard M-AMBI framework, the Bad class represents an azoic condition (AMBI = 6, richness = 0 and Shannon diversity = 0), whereas the High class is defined from high/reference values of AMBI, richness and Shannon diversity (Muxika, Borja & Bald, 2007; Borja & Dauer, 2008).

Environmental associations were evaluated using the subset containing 30 stations: 19 from the Vitória Bay (VB + CP) and 11 from the Piraquê-Açu–Mirim group (PA + PM). Macrofaunal abundance, diversity and ecological-status analysis used the complete macrofaunal or AMBI/M-AMBI dataset. Analyses linking community structure and ecological metrics to environmental variables (sediment geochemistry and contaminants) were restricted to the 30 stations sampled for sediment organic geochemistry. Macrofaunal abundances were square-root transformed, Bray–Curtis resemblance matrices were calculated (Bray & Curtis, 1957), environmental predictors were log(X + 1) transformed. The environmental dataset included salinity, BPC, fecal sterols, total sterols, Cr, Zn, Fe, Al, TOC, TN and % mud.

Before multivariate analyses, the environmental matrix was screened for missing and invalid values. One invalid record was identified: the Pb concentration at station PA10 was negative. Because no laboratory-verified replacement sample was available, the record was not imputed, and Pb was therefore excluded as a predictor from all reported environmental analyses (DistLM marginal tests, dbRDA/CAP and Pearson correlations), while station PA10 was retained using its remaining valid environmental measurements. Group-specific DistLM analyses were performed separately for VB + CP (*n* = 19) and PA + PM (*n* = 11) in PRIMER v7 with the PERMANOVA+ add-on (Anderson, Gorley & Clarke, 2008; Clarke & Gorley, 2015). DistLM marginal tests had their environmental predictors fitted separately to the Bray–Curtis community resemblance matrix; the reported quantities are the marginal proportion of variation, pseudo-F statistic and permutation *p*-value for that one-predictor test. The PRIMER BEST procedure with the unadjusted R^2^ criterion was used as exploratory model-selection output. Because the number of candidate predictors was large relative to the group-specific sample sizes, particularly for PA + PM, no sequential multi-predictor DistLM model was used. BEST model selection followed the BIO-ENV/BEST framework of Clarke & Ainsworth (1993). DistLM analysis used here did not include an explicit spatial covariance term or a spatially restricted permutation scheme, so it does not explicitly account for spatial autocorrelation. Spatial dependence can violate the independence/exchangeability assumptions underlying standard permutation inference (Legendre, 1993; Anderson, Gorley & Clarke, 2008); consequently, the DistLM results are interpreted here as exploratory spatial associations rather than as spatially independent or causal effects.

A principal component analysis (PCA) was used for descriptive visualization of the environmental matrix across stations sampled and to help determine reference conditions (non-polluted sites). The same log(X + 1)-transformed data were used to summarize covariation among environmental measurements. A distance-based redundancy analysis/canonical analysis of principal coordinates (dbRDA/CAP) was fitted to Bray-Curtis dissimilarities with an additive correction for negative eigenvalues using capscale in the vegan package (Legendre & Anderson, 1999; Oksanen et al., 2026). Valid environmental data were log(X + 1)-transformed and standardized before fitting. Environmental arrows represent correlations between predictors and site scores, and only the eight strongest axis correlations were displayed. Dotted ellipses summarize the two environmental-analysis groups.

Pearson correlations were calculated across the environmental dataset after transformation log(X + 1) and compared with four benthic assemblage variables: abundance density, taxonomic richness, AMBI and M-AMBI (Pearson, 1895). For these correlations, two-sided raw *p*-values were obtained and adjusted with the Benjamini–Hochberg method (Benjamini & Hochberg, 1995). Statistical analyses were performed in R (R Core Team, 2020) using readxl, dplyr, tidyr, vegan, ggplot2, viridis and patchwork.

## Results

### Macrofaunal abundance, diversity and composition

A total of 27,502 macrofaunal individuals representing 168 benthic metazoan taxa were recorded across the 64 sampling stations. Vitória Bay, including the Canal da Passagem (CP) channel, contained 19,070 individuals with a total of 168 taxa recorded. The Piraquê-Açu estuary contained 4,998 individuals and 94 taxa, whereas Piraquê-Mirim contained 3,434 individuals and 74 taxa (Figs. S1, S2). Macrofaunal abundance ranged from 75 to 12,980 individuals m^−2^, with the highest values recorded in the inner estuarine sector of Vitória Bay, particularly at VB12. This station also showed the highest taxonomic richness and diversity, with 74 taxa, Shannon diversity of H′ = 4.96 and relatively high evenness (J′ = 0.80; Fig. S2). Other inner Vitória Bay stations, including VB09 to VB15, showed similar patterns, characterized by relatively high richness, diversity and more even assemblages in this sector.

In contrast, the Canal da Passagem (CP) stations showed reduced abundance and diversity, with assemblages frequently dominated by oligochaetes and capitellid polychaetes. This pattern indicates a more simplified community structure, consistent with localized environmental stress and strong dominance by tolerant or opportunistic taxa. The outer marine-influenced Vitória Bay stations, particularly VB23 to VB36, also exhibited low macrofaunal densities, but generally retained slightly higher taxonomic richness than the CP stations, suggesting that low abundance in this sector was not necessarily associated with the same degree of dominance observed in the channel. Overall, Vitória Bay displayed strong spatial heterogeneity, with diverse and relatively even assemblages in the inner estuarine sector, species-poor assemblages with high dominance in the CP, and low-density but comparatively richer assemblages in the outer bay. In the Piraquê-Açu–Mirim estuary, several Piraquê-Mirim stations had species-poor assemblages. Despite these spatial differences, the overall mean macrofaunal abundances were relatively similar: 1,540 individuals m^−2^ in Vitória Bay, 1,677 individuals m^−2^ in Piraquê-Açu and 1,480 individuals m^−2^ in Piraquê-Mirim.

Macrofaunal community composition differed among estuaries and stations (Fig. 2). Oligochaeta was the most abundant taxon, representing 9.6% of all individuals sampled. Other broadly abundant taxa included the polychaetes *Mediomastus* sp. (5.9%), *Tharyx* sp. (5.4%) and *Lumbrineris* sp. (5.3%), together with bivalves and decapod crustaceans that occurred locally across the study area. Vitória Bay was characterized by a broader dominance, with oligochaeta as the dominant taxon (13.7%), followed by *Mediomastus* sp. (8.2%), *Tagelus divisus* (4.5%) and Tharyx sp. (4.4%). At VB17, oligochaeta comprised 75.7% of the pooled assemblage, with low Shannon diversity (H′ = 1.74) and evenness (J′ = 0.37). In the VB channel, CP04 contained only 15 individuals dominated by oligochaeta and Capitella sp., which together represented 60% of the recorded individuals. All other stations in the passage channel (CP01-08) exhibited a similar low diversity and high dominance pattern (Fig. 2). In contrast, the Piraquê-Açu estuary was mainly dominated by *Lumbrineris* sp. (13.0%), *Magelona papillicornis* (11.5%) and *Tharyx* sp. (10.3%), whereas Piraquê-Mirim showed a more concentrated dominance led by *Lumbrineris* sp. (19.2%), *Gymnonereis crosslandi* (16.5%) and *Dorvillea* sp. (11.3%). These patterns indicate clear differences in the dominant macrofaunal taxa among sectors, with Vitória Bay showing a more diversified dominance structure and Piraquê-Mirim showing stronger concentration of abundance in fewer taxa (Fig. S2).

**Figure 2:**
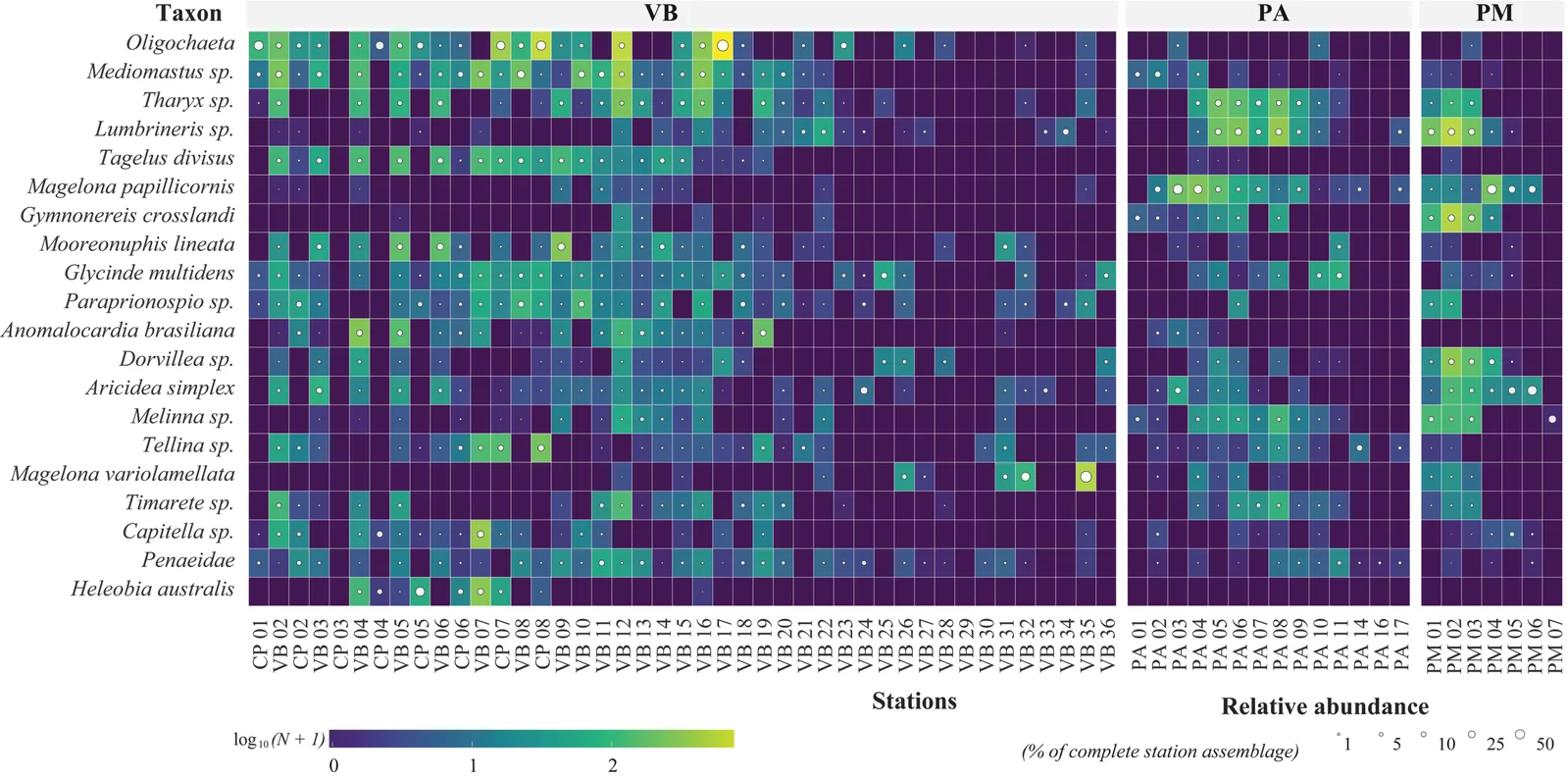
Benthic composition and abundance. Macrofaunal composition and abundance of the top-20 ranked taxa across study areas in Vitória Bay (VB), Piraquê-Açu (PA) and Piraquê-Mirim (PM) estuaries. Tile color represents station-level log_10_(N + 1) abundance, and bubble area represents each taxon’s relative abundance as a percentage of the station’s total. Stations are grouped by estuary; VB (Vitoria Bay), PA (Piraquê-Açu) and PM (Piraquê-Mirim).

### AMBI and M-AMBI ecological status

In Vitória Bay, AMBI ranged from 1.0 at VB36 to 5.1 at VB17, whereas stations within the Canal da Passagem ranged from 2.5 to 6.0, with the highest disturbance at CP04 (Fig. 3, Table S2). Most VB stations were classified as undisturbed to slightly disturbed according to AMBI, broadly consistent with M-AMBI classifications of High, Good or Moderate ecological status. In contrast, several CP stations and VB17 were classified as Poor or Bad by M-AMBI. In the PAM estuary, most PA and PM stations exhibited High or Good ecological status and undisturbed or slightly disturbed AMBI conditions. The main exception was PM07, which showed Bad ecological status and a heavily disturbed AMBI condition. Overall, both indices indicated strong spatial heterogeneity rather than uniform degradation across entire estuarine sectors (Fig. 3; Table S2, Figs. S3, S4).

**Figure 3:**
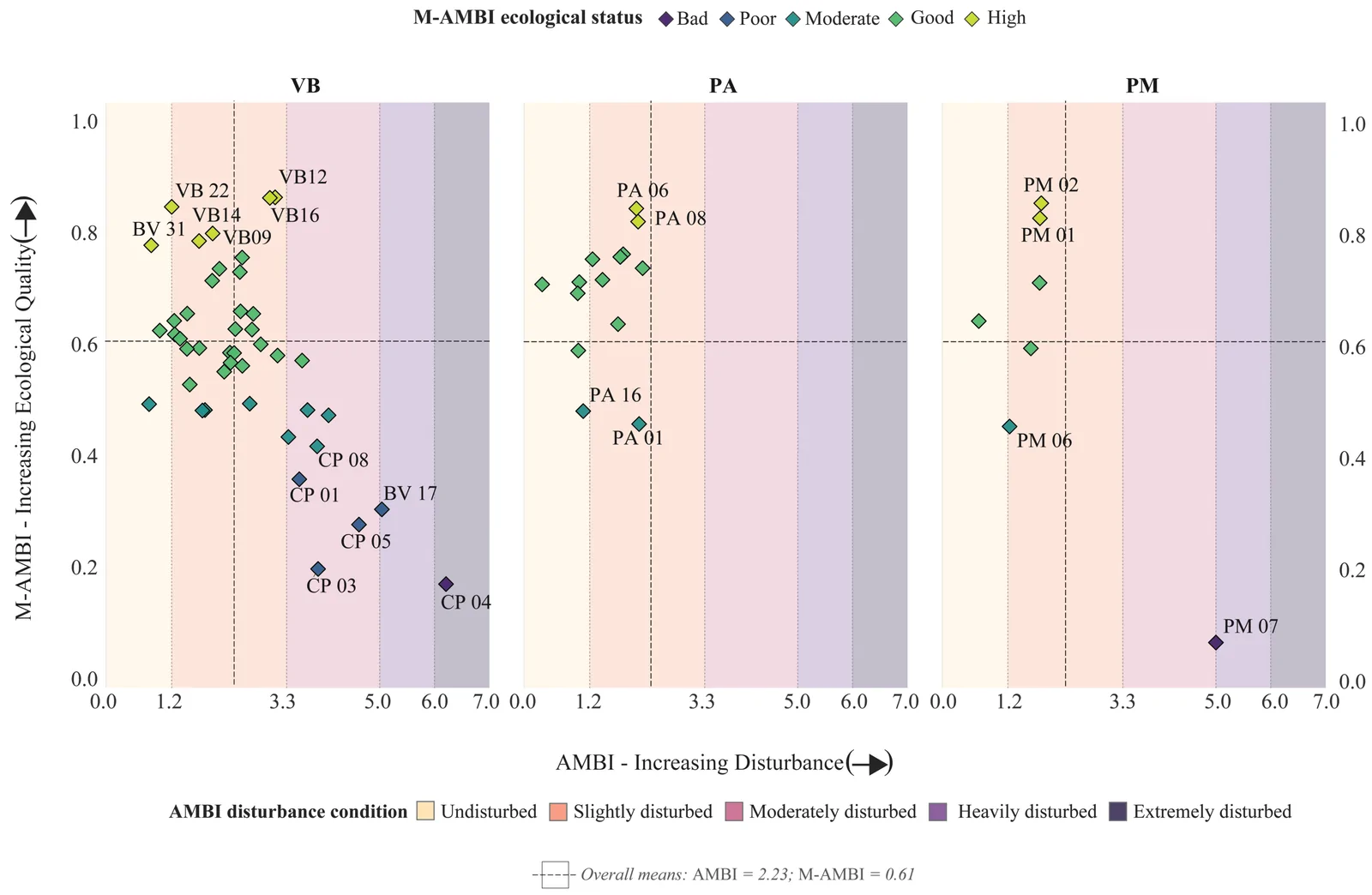
Benthic biotic indices from sampled sites. Benthic ecological quality indices in Vitória Bay (VB), Piraquê-Açu (PA) and Piraquê-Mirim (PM) estuaries. Station-level AMBI values are plotted against M-AMBI. Background bands show AMBI disturbance classes: Undisturbed (AMBI ≤ 1.2), Slightly disturbed (>1.2–3.3), Moderately disturbed (>3.3–5.0), Heavily disturbed (>5.0–6.0) and Extremely disturbed (>6.0). Point colors indicate M-AMBI ecological status (Bad, Poor, Moderate, Good or High). Dashed lines show the overall mean AMBI and M-AMBI values.

### Environmental gradients in macrofaunal community structure

The environmental and benthic macrofaunal assemblage ordination revealed a spatial separation aligned with gradients in salinity and sedimentary sterols, including fecal sterols (Fig. 4; Figs. S5; S6). The first dbRDA/CAP axis represented 10.2% of total community inertia and the second represented 8.6%. Positive scores on the first axis were associated primarily with increasing salinity and included several outer Vitória Bay stations, particularly VB24, VB27, VB30, VB32, VB34 and VB36. Negative scores were associated with higher fecal sterols, total sterols and biopolymeric carbon and included most Canal da Passagem stations, together with VB17 and VB19. The second axis indicated environmental differences among Piraquê-Açu and Piraquê-Mirim stations, mostly along gradients of sedimentary organic matter, fine sediments and trace elements. Negative scores on this axis were associated with total organic carbon, percentage of mud, Cr and Fe. PA05, PA07, PA08, PA09, PA10, PM01 and PM06 occurred predominantly in this portion of the ordination. Several VB stations were also associated with the sterol gradient, particularly CP04, CP06 and CP07 (Fig. 4).

**Figure 4:**
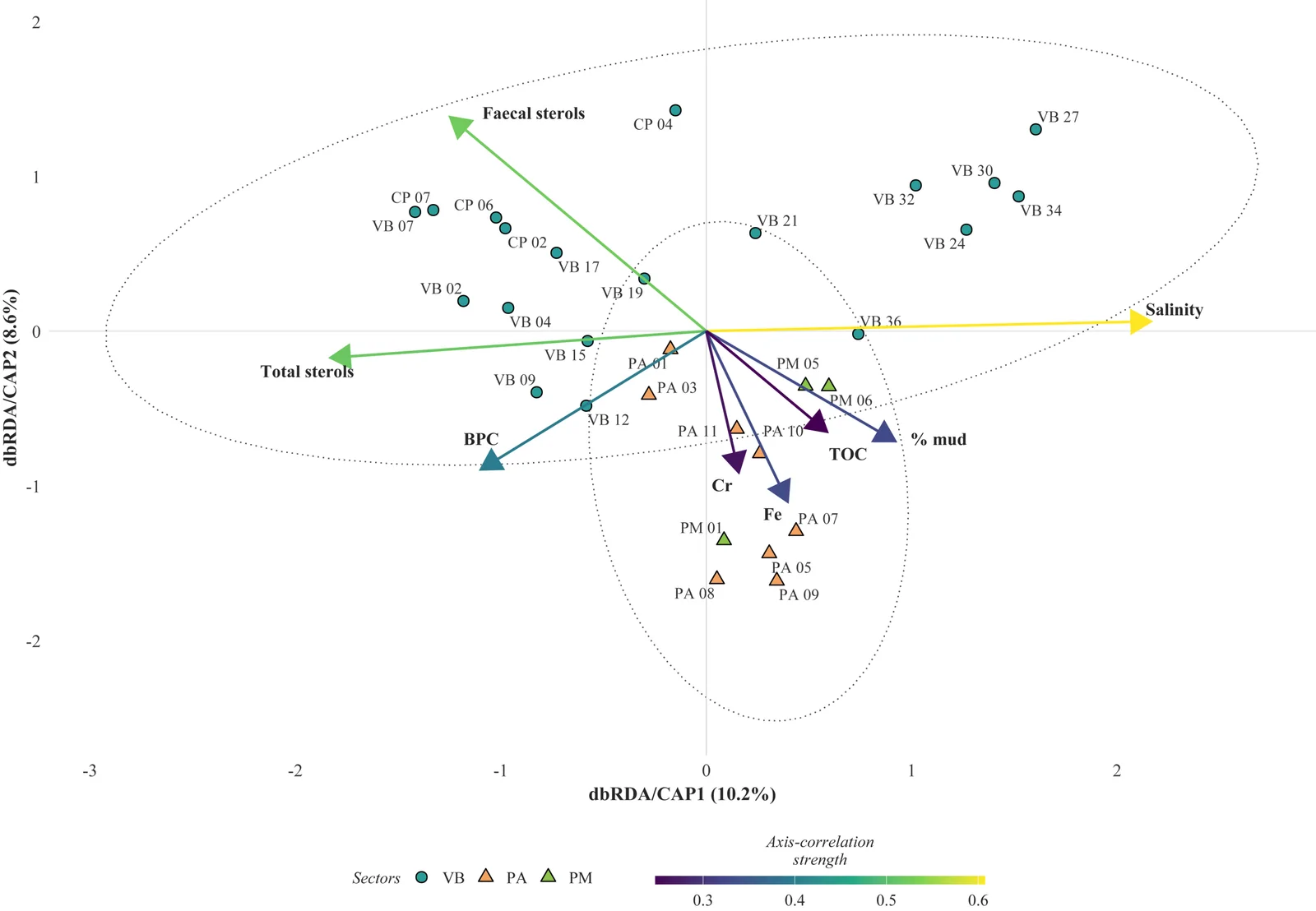
Ordination analysis of benthic and environmental variables. Combined dbRDA/CAP ordination of macrofaunal abundances based on Bray–Curtis dissimilarities. Axis labels indicate the percentage of total community inertia represented by each axis. Arrows show the eight strongest correlations between valid environmental data and station benthic assemblages, with arrow colors indicating correlation strength. Dotted ellipses summarize the Vitória Bay (VB + CP) and Piraquê-Açu–Mirim (PA + PM) groups.

Group-specific Distance-based Linear Model marginal tests showed contrasting patterns between the two estuarine systems (Fig. 5A). In Vitória Bay (VB + CP; *n* = 19), salinity had the largest marginal association with community variation, accounting for 15.3% of the fitted assemblage variance (pseudo-F = 3.061, nominal permutation *p* < 0.05). Additional marginal associations with nominal, uncorrected permutation *p* < 0.05 were observed for total sterols (11.9%; pseudo-F = 2.296, *p* < 0.05) and biopolymeric carbon (BPC; 10.1%; pseudo-F = 1.904, *p* < 0.05). Other predictors had lower marginal contributions, including Fe (7.1%), Al (6.4%), Cr (6.2%), Zn (6.1%), % mud (6.0%), faecal sterols (5.6%) and TOC (5.2%).

**Figure 5:**
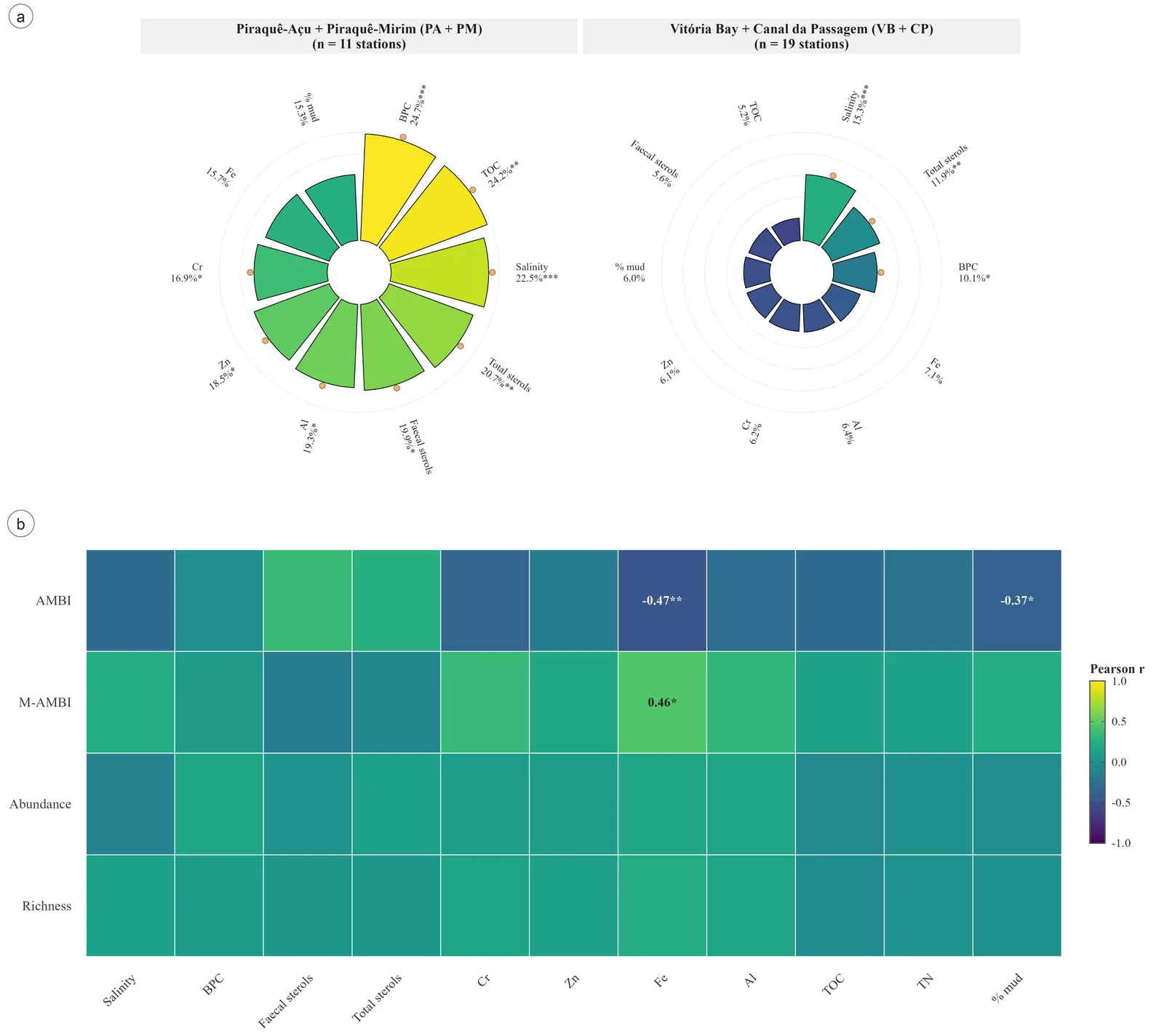
Correspondence analysis between benthic indices and environmental variables. Environmental associations with benthic ecological indices. (A) Circular bar plots show DistLM marginal tests for the Piraquê-Açu–Mirim estuary (PA + PM) and Vitória Bay (VB + CP). Bar length and color represent the marginal proportion of community variation associated with each predictor when fitted separately. Orange dots denote significant marginal tests (*p ≤ 0.05; **p ≤ 0.01; ***p ≤ 0.001). (B) Pearson correlations between AMBI, M-AMBI, abundance and richness across sites. Cell color represents the Pearson r.

In the Piraquê-Açu–Mirim estuary (PA + PM; *n* = 11), benthic assemblages were most strongly associated with TN (24.9%), BPC (24.7%), TOC (24.2%) and salinity (22.5%). Total sterols (20.7%) and fecal sterols (19.9%) also showed strong marginal associations, together with sedimentary elements including Al (19.3%), Zn (18.5%) and Cr (16.9%). Fe (15.7%) and % mud (15.3%) also contributed to community variation but had smaller marginal contributions. The marginal pattern suggests that community variation in the Piraquê-Açu–Mirim system was structured by a combination of organic-matter availability, salinity, sediment texture and geochemical gradients.

Pearson correlations between ecological indices and environmental predictors identified three nominal associations: AMBI was negatively correlated with Fe (*r* = −0.473) and percentage of mud (*r* = −0.374), whereas M-AMBI was positively correlated with Fe (*r* = 0.455; Fig. 5B). However, none of the correlations remained significant after Benjamini–Hochberg FDR correction (all *q* > 0.05). This result indicates that AMBI and M-AMBI were not directly related to the measured sediment geochemical data after accounting for multiple testing. The nominal relationships involving Fe and mud may reflect depositional and grain-size gradients as well as potential local enrichment.

## Discussion

Our study showed that benthic macrofaunal assemblages are strongly structured by sedimentary organic-matter quality, sediment contamination and salinity gradients across two contrasting estuaries in SE Brazil. Even in the heavily urbanized Vitória Bay estuary, the integration of benthic assemblages with ecological indices revealed that anthropogenic impacts were spatially localized. In general, AMBI and M-AMBI highlighted areas of both high and low ecological quality, with the spatial variability coupled to taxa and geochemical markers that are typically associated to polluted sediments. As a result, our study supports the interpretation that ecological status in tropical estuaries is best understood as a spatial mosaic rather than as a uniform response to urbanization or eutrophication.

We observed spatially dependent impacts on benthic assemblages in both estuaries, indicating contrasting pollution levels. Areas with high ecological quality occurred near organically polluted sites, supporting previous contamination assessments in this region (Hadlich et al., 2018). In addition, benthic assemblages also indicated poor-condition status outside the estuarine areas that were not directly associated with contamination, which could suggest reduced sensitivity of these indices when individual counts are low (Muniz et al., 2005). Although we detected a marked heterogeneity of sediment quality in both estuaries, both indices overall performed well and were consistent with site-level contaminant sources and biological transitions over short distances (Dauvin, 2007; Elliott & Quintino, 2007; Borja & Dauer, 2008).

We found that estuarine assemblages are also significantly structured by natural gradients of salinity and sediment quality. Salinity and grain size were significant drivers of benthic assemblage associations in both estuaries, even though they were embedded within a patchy contamination background. Salinity is commonly a dominant determinant of estuarine macrofaunal composition because it constrains osmoregulation, recruitment, species distributions and the size of the regional species pool (Alves, Petsch & Barros, 2020). Its ecological effect may exceed or mask pollutant responses when sites span mesohaline to polyhaline conditions (Elliott & Quintino, 2007; Borja & Tunberg, 2011). This combination underlies the “estuarine quality paradox” in which biological characteristics normally associated with pollution may partly reflect natural habitat variability (Dauvin, 2007; Elliott & Quintino, 2007). At the most degraded stations, the concentration of abundance in few tolerant or opportunistic taxa is consistent with the organic-enrichment succession model proposed by Pearson & Rosenberg (1978). Under increasing enrichment, diverse assemblages containing sensitive and long-lived species are progressively replaced by small-bodied, rapidly reproducing deposit feeders and opportunists; when enrichment is accompanied by oxygen depletion, diversity and functional complexity may decline further (Gray, Wu & Or, 2002; Diaz & Rosenberg, 2008).

The association of community variation with biopolymeric carbon, total organic carbon, total nitrogen and sterols points to the importance of sedimentary organic-matter quality to benthic assemblages. Biopolymeric carbon integrates the protein, carbohydrate and lipid fractions of sedimentary organic matter and has been used to distinguish trophic conditions and the fraction of organic matter more readily available to benthic consumers and microbes (Fabiano & Danovaro, 1994; Pusceddu et al., 2007). High organic availability may initially support greater benthic production, but persistent loading can enhance microbial oxygen demand, alter redox conditions and favor tolerant deposit feeders and opportunists (Pearson & Rosenberg, 1978; Gray, Wu & Or, 2002).

We also observed differences between benthic assemblages potentially exposed to direct and indirect effects of organic pollutants in both estuaries. AMBI was explicitly driven by the relative abundance of ecological groups that indicated organic-enrichment and disturbance gradients, especially in Vitória Bay. AMBI also indicated variable ecological status in the pristine PAM estuary, revealing its sensitivity to localized pollution sources. M-AMBI complemented its sensitivity by integrating richness and Shannon diversity with AMBI, thereby distinguishing stations that may have similar ecological-group composition but different levels of taxonomic complexity (Muxika, Borja & Bald, 2007; Borja et al., 2008).

Comparable spatial differences in these indices have been reported worldwide, with AMBI and M-AMBI successfully detected stress across subtropical estuaries (Borja & Tunberg, 2011). Previous studies suggested that regional calibration of M-AMBI improved concordance between indices and reduced bias associated with natural environmental gradients (Pelletier et al., 2018; Muniz et al., 2005). Although we did not detect statistically significant relationships between indices and organic contaminants, our indices performed well across areas with marked differences in sediment pollution (Checon et al., 2018). This suggests that increased monitoring and regional taxonomic assignments would markedly increase the capacity of these indices to detect impacts, as potential seasonal variability in assemblages would be detected (Mulik, Sukumaran & Dias, 2020).

This study further supports the applicability of AMBI outside Europe, despite the ecological-group database having been developed largely from temperate fauna. Nevertheless, testing in the South Atlantic demonstrated that AMBI can be further improved when taxonomic assignments are carefully evaluated (Muniz et al., 2005; Valença & Santos, 2012; Checon et al., 2018). A subtropical Brazilian comparison further showed that AMBI, M-AMBI and BENTIX can differ in sensitivity to hydrodynamic and sewage-related gradients (Brauko et al., 2015). The growing global application of AMBI has confirmed its utility across multiple regions and pressures, but has also reinforced the need for regional taxonomic assignments (Borja, Dauer & Grémare, 2012; Borja, Chust & Muxika, 2019).

The present study advances the use of ecological indices in urban and pristine tropical estuaries in Brazil, and supports their applicability to detect short-scale contamination patchiness and ecological status, both of which are relevant to coastal management. The combined use of AMBI and M-AMBI indices and graphical outputs is more explicit and user-friendly than benthic macrofaunal assemblage descriptors alone. The coexistence of high-quality stations and localized degradation within estuaries has strong implications for monitoring sampling designs, but can also indicate potential differences in ecosystem processes and services. Considering that benthic assemblages are critical to overall ecosystem health and services quantifying economic and social losses due to estuarine habitat degradation would be an important step to aid management. These steps would aid in broader and more holistic ecosystem services valuation by estuaries (Barbier et al., 2011; Owuor et al., 2024; Otieno et al., 2026), with direct management recommendations to address pollutant sources and land use change of critical habitats including mangroves and salt marshes.

## Conclusions

Our results showed spatially heterogeneous sediment ecological quality in both the urban and non-urban estuaries in tropical Brazil. Benthic ecological health status revealed patchy effects of contamination and the combined interpretation of macrofaunal structure, AMBI, M-AMBI and sedimentary indicators was effective in identifying both localized degradation and the broader natural salinity gradients. Our study further supports the use of benthic indices in urbanized estuaries and supports its use to identify areas of ecological value within tropical estuaries.

## Supplemental Information

10.7717/peerj.21883/supp-1 Supplemental Information 1 Supplementary tables and figures

## Additional Information and Declarations

### Field Study Permissions

The following information was supplied relating to field study approvals (i.e., approving body and any reference numbers): Field experiments were approved by the Instituto Chico Mendes da Conservação da Biodiversidade (ICMBIo).

### Data Availability

The following information was supplied regarding data availability: The raw biodiversity data are available in the supplementary files and are available at the Brazilian Biodiversity Repository (Bernardino & Anderson, 2026): - Bernardino A, Batista A (2026). PELD - HCES — Estuarine macrofauna from Vitoria Bay and Piraque-Acu-Mirim estuaries. Version 2.0. Universidade Federal do Espírito Santo. Samplingevent dataset. https://doi.org/10.71819/w8wfir
