# Supplementary material for: Contrasting benthic ecological quality within tropical estuaries in Southeastern Brazil based on biotic indices
Source: PeerJ. 2026 Oct 22;14:e21883. doi: 10.7717/peerj.21883 (PMC13695960; doi:10.7717/peerj.21883)
Supplement: Supplementary file 1 [file peerj-14-21883-s001.docx]

**Supplementary Material for: Contrasting benthic ecological quality within tropical estuaries in SE Brazil based on biotic indices**

**Supplementary Table S1.** Ecological-group assignments of benthic taxa sampled in this study and used to calculate the AMBI indices. 1. Indicates adjustments made in taxa assignment. Unassigned taxa (N=39) were not considered in AMBI calculation.

| **No.** | **Major group** | **Taxon ID** | **AMBI V5.0 ID** | **AMBI V5.0 group** | **Ecological interpretation** | **Adjustment^1^** | **Notes** |
| --- | --- | --- | --- | --- | --- | --- | --- |
| 1 | Oligochaeta | Oligochaeta | Oligochaeta | V | First-order opportunists | Exact | Exact taxon name in current AMBI list. |
| 2 | Echiura | Echiura | Echiura | II | Indifferent taxa | Exact | Exact taxon name in current AMBI list. |
| 3 | Polychaeta | *Isolda pulchella* | *Isolda pulchella* | III | Tolerant taxa | Exact | Exact taxon name in current AMBI list. |
| 4 | Polychaeta | *Melinna sp.* | *Melinna sp.* | III | Tolerant taxa | Exact | Exact taxon name in current AMBI list. |
| 5 | Polychaeta | *Capitella sp.* | *Capitella sp.* | V | First-order opportunists | Exact | Exact taxon name in current AMBI list. |
| 6 | Polychaeta | *Heteromastus sp.* | *Heteromastus sp.* | IV | Second-order opportunists | Exact | Exact taxon name in current AMBI list. |
| 7 | Polychaeta | *Mediomastus sp.* | *Mediomastus sp.* | III | Tolerant taxa | Exact | Exact taxon name in current AMBI list. |
| 8 | Polychaeta | *Spiochaetopterus sp.* | *Spiochaetopterus sp.* | III | Tolerant taxa | Exact | Exact taxon name in current AMBI list. |
| 9 | Polychaeta | Cirratulidae | Cirratulidae | IV | Second-order opportunists | Exact | Exact taxon name in current AMBI list. |
| 10 | Polychaeta | *Aphelochaeta sp.* | *Aphelochaeta sp.* | IV | Second-order opportunists | Exact | Exact taxon name in current AMBI list. |
| 11 | Polychaeta | *Tharyx sp.* | *Tharyx sp.* | IV | Second-order opportunists | Exact | Exact taxon name in current AMBI list. |
| 12 | Polychaeta | *Chaetozone sp.* | *Chaetozone sp.* | IV | Second-order opportunists | Exact | Exact taxon name in current AMBI list. |
| 13 | Polychaeta | *Timarete sp.* | *Timarete sp.* | IV | Second-order opportunists | Exact | Exact taxon name in current AMBI list. |
| 14 | Polychaeta | *Cossura sp.* | *Cossura sp.* | IV | Second-order opportunists | Exact | Exact taxon name in current AMBI list. |
| 15 | Polychaeta | *Dorvillea sp.* | *Dorvillea sp.* |  | Unassigned | Unassigned | Taxa unassigned |
| 16 | Polychaeta | *Eunice sp.* | *Eunice sp.* | II | Indifferent taxa | Exact | Exact taxon name in current AMBI list. |
| 17 | Polychaeta | *Lysidice sp.* | *Lysidice sp.* | II | Indifferent taxa | Exact | Exact taxon name in current AMBI list. |
| 18 | Polychaeta | *Marphysa sp* | *Marphysa sp.* | II | Indifferent taxa | Exact | Exact taxon name in current AMBI list. |
| 19 | Polychaeta | *Nematonereis sp.* | *Nematonereis sp.* | II | Indifferent taxa | Exact | Exact taxon name in current AMBI list. |
| 20 | Polychaeta | *Pherusa sp.* | *Pherusa sp.* | I | Sensitive taxa | Exact | Exact taxon name in current AMBI list. |
| 21 | Polychaeta | *Piromis sp.* | *Piromis sp.* | I | Sensitive taxa | Exact | Exact taxon name in current AMBI list. |
| 22 | Polychaeta | *Glycera americana* | *Glycera americana* | I | Sensitive taxa | Exact | Exact taxon name in current AMBI list. |
| 23 | Polychaeta | *Goniadidae ñ id.* | Goniadidae | II | Indifferent taxa | Exact | Exact taxon name in current AMBI list. |
| 24 | Polychaeta | *Goniada sp.* | *Goniada sp.* | II | Indifferent taxa | Exact | Exact taxon name in current AMBI list. |
| 25 | Polychaeta | *Glycinde multidens* | *Glycinde multidens* | II | Indifferent taxa | Exact | Exact taxon name in current AMBI list. |
| 26 | Polychaeta | *Hesione sp.* | *Hesione sp.* | II | Indifferent taxa | Exact | Exact taxon name in current AMBI list. |
| 27 | Polychaeta | *Gyptis sp.* | *Gyptis sp.* | II | Indifferent taxa | Exact | Exact taxon name in current AMBI list. |
| 28 | Polychaeta | *Periboea sp.* | — |  | Unassigned | Unassigned | Taxa unassigned |
| 29 | Polychaeta | *Lumbrineris sp.* | *Lumbrineris sp.* | II | Indifferent taxa | Exact | Exact taxon name in current AMBI list. |
| 30 | Polychaeta | *Magelona papillicornis* | *Magelona papillicornis* | I | Sensitive taxa | Exact | Exact taxon name in current AMBI list. |
| 31 | Polychaeta | *Magelona variolamellata* | *Magelona variolamellata* | I | Sensitive taxa | Exact | Exact taxon name in current AMBI list. |
| 32 | Polychaeta | *Magelona sp.* | *Magelona sp.* | I | Sensitive taxa | Exact | Exact taxon name in current AMBI list. |
| 33 | Polychaeta | Maldanidae | Maldanidae | I | Sensitive taxa | Exact | Exact taxon name in current AMBI list. |
| 34 | Polychaeta | *Axiothella sp.* | *Axiothella sp.* | I | Sensitive taxa | Exact | Exact taxon name in current AMBI list. |
| 35 | Polychaeta | *Clymenella sp.* | *Clymenella sp.* | I | Sensitive taxa | Exact | Exact taxon name in current AMBI list. |
| 36 | Polychaeta | *Euclymene sp.* | *Euclymene sp.* |  | Unassigned | Unassigned | Taxa unassigned |
| 37 | Polychaeta | *Nephtys sp.* | *Nephtys sp.* | II | Indifferent taxa | Exact | Exact taxon name in current AMBI list. |
| 38 | Polychaeta | Nereididae | Nereididae |  | Unassigned | Unassigned | Taxa unassigned |
| 39 | Polychaeta | *Gymnonereis crosslandi* | *Gymnonereis crosslandi* | II | Indifferent taxa | Exact | Exact taxon name in current AMBI list. |
| 40 | Polychaeta | *Laeonereis culveri* | *Laeonereis culveri* | IV | Second-order opportunists | Exact | Exact taxon name in current AMBI list. |
| 41 | Polychaeta | *Neanthes succinea* | *Neanthes succinea* | III | Tolerant taxa | Exact | Exact taxon name in current AMBI list. |
| 42 | Polychaeta | *Neanthes bruaca* | *Neanthes bruaca* | III | Tolerant taxa | Exact | Exact taxon name in current AMBI list. |
| 43 | Polychaeta | *Perinereis sp.* | *Perinereis sp.* | III | Tolerant taxa | Exact | Exact taxon name in current AMBI list. |
| 44 | Polychaeta | Oenonidae | — |  | Unassigned | Unassigned | Taxa unassigned |
| 45 | Polychaeta | *Onuphidae ñ id.* | Onuphidae | II | Indifferent taxa | Exact | Exact taxon name in current AMBI list |
| 46 | Polychaeta | *Diopatra cuprea* | *Diopatra cuprea* | I | Sensitive taxa | Exact | Exact taxon name in current AMBI list. |
| 47 | Polychaeta | *Kinbergonuphis sp.* | *Kinbergonuphis sp.* | II | Indifferent taxa | Exact | Exact taxon name in current AMBI list. |
| 48 | Polychaeta | *Mooreonuphis lineata* | *Mooreonuphis lineata* | II | Indifferent taxa | Exact | Exact taxon name in current AMBI list. |
| 49 | Polychaeta | *Onuphis eremita* | *Onuphis eremita* | II | Indifferent taxa | Exact | Exact taxon name in current AMBI list. |
| 50 | Polychaeta | *Ophelina sp.* | *Ophelina sp.* |  | Unassigned | Unassigned | Taxa unassigned |
| 51 | Polychaeta | *Haploscoloplos sp.* | *Haploscoloplos sp.* | III | Tolerant taxa | Exact | Exact taxon name in current AMBI list. |
| 52 | Polychaeta | *Leitoscoloplos sp.* | *Leitoscoloplos sp.* | IV | Second-order opportunists | Exact | Exact taxon name in current AMBI list. |
| 53 | Polychaeta | *Orbinia sp.* | *Orbinia sp.* | I | Sensitive taxa | Exact | Exact taxon name in current AMBI list. |
| 54 | Polychaeta | *Scoloplos sp.* | *Scoloplos sp.* | I | Sensitive taxa | Exact | Exact taxon name in current AMBI list. |
| 55 | Polychaeta | *Naineris setosa* | *Naineris setosa* | I | Sensitive taxa | Exact | Exact taxon name in current AMBI list. |
| 56 | Polychaeta | *Galathowenia oculata* | *Galathowenia oculata* | II | Indifferent taxa | Exact | Exact taxon name in current AMBI list. |
| 57 | Polychaeta | *Owenia sp.* | — |  | Unassigned | Unassigned | Taxa unassigned |
| 58 | Polychaeta | *Paraonidae ñ id.* | Paraonidae | II | Indifferent taxa | Exact | Exact taxon name in current AMBI list. |
| 59 | Polychaeta | *Aricidea simplex* | *Aricidea simplex* | II | Indifferent taxa | Exact | Exact taxon name in current AMBI list. |
| 60 | Polychaeta | *Cistenides sp.* | *Cistenides sp.* | I | Sensitive taxa | Exact | Exact taxon name in current AMBI list. |
| 61 | Polychaeta | *Phyllodocidae ñ id.* | Phyllodocidae | II | Indifferent taxa | Exact | Exact taxon name in current AMBI list. |
| 62 | Polychaeta | *Eumida sp.* | *Eumida sp.* | II | Indifferent taxa | Exact | Exact taxon name in current AMBI list. |
| 63 | Polychaeta | *Phyllodoce sp.* | *Phyllodoce sp.* | II | Indifferent taxa | Exact | Exact taxon name in current AMBI list. |
| 64 | Polychaeta | *Ancistrosyllis sp.* | *Ancistrosyllis sp.* | III | Tolerant taxa | Exact | Exact taxon name in current AMBI list. |
| 65 | Polychaeta | *Loandalia sp.* | — |  | Unassigned | Unassigned | Taxa unassigned |
| 66 | Polychaeta | *Sigambra grubii* | *Sigambra grubii* | IV | Second-order opportunists | Exact | Exact taxon name in current AMBI list. |
| 67 | Polychaeta | *Poecilochaetus sp.* | *Poecilochaetus sp.* | I | Sensitive taxa | Exact | Exact taxon name in current AMBI list. |
| 68 | Polychaeta | *Eunoe serrata* | *Eunoe serrata* | II | Indifferent taxa | Exact | Exact taxon name in current AMBI list. |
| 69 | Polychaeta | *Eunoe papilosa* | — |  | Unassigned | Unassigned | Taxa unassigned |
| 70 | Polychaeta | *Harmothoe aculeata* | *Harmothoe aculeata* | II | Indifferent taxa | Exact | Exact taxon name in current AMBI list. |
| 71 | Polychaeta | *Psionidens sp.* | — |  | Unassigned | Unassigned | Taxa unassigned |
| 72 | Polychaeta | *Sabellidae ñ id.* | Sabellidae | I | Sensitive taxa | Exact | Exact taxon name in current AMBI list. |
| 73 | Polychaeta | *Chone sp.* | *Chone sp.* | II | Indifferent taxa | Exact | Exact taxon name in current AMBI list. |
| 74 | Polychaeta | *Hypsicomus sp.* | — |  | Unassigned | Unassigned | Taxa unassigned |
| 75 | Polychaeta | *Megalomma sp.* | *Megalomma sp.* | II | Indifferent taxa | Exact | Exact taxon name in current AMBI list. |
| 76 | Polychaeta | *Sabella sp.* | *Sabella sp.* | I | Sensitive taxa | Exact | Exact taxon name in current AMBI list. |
| 77 | Polychaeta | *Sigalion sp.* | *Sigalion sp.* | II | Indifferent taxa | Exact | Exact taxon name in current AMBI list. |
| 78 | Polychaeta | *Sthenelais sp.* | *Sthenelais sp.* | II | Indifferent taxa | Exact | Exact taxon name in current AMBI list. |
| 79 | Polychaeta | Spionidae | Spionidae | III | Tolerant taxa | Exact | Exact taxon name in current AMBI list. |
| 80 | Polychaeta | *Microspio pigmentata* | *Microspio pigmentata* | II | Indifferent taxa | Exact | Exact taxon name in current AMBI list. |
| 81 | Polychaeta | *Laonice sp.* | *Laonice sp.* | III | Tolerant taxa | Exact | Exact taxon name in current AMBI list. |
| 82 | Polychaeta | *Paraprionospio sp.* | *Paraprionospio sp.* | IV | Second-order opportunists | Exact | Exact taxon name in current AMBI list. |
| 83 | Polychaeta | *Prionospio sp.* | *Prionospio sp.* |  | Unassigned | Unassigned | Taxa unassigned |
| 84 | Polychaeta | *Polydora cornuta* | *Polydora cornuta* | IV | Second-order opportunists | Exact | Exact taxon name in current AMBI list. |
| 85 | Polychaeta | *Polydora nuchalis* | *Polydora nuchalis* | III | Tolerant taxa | Exact | Exact taxon name in current AMBI list. |
| 86 | Polychaeta | *Polydora sp.* | *Polydora sp.* | IV | Second-order opportunists | Exact | Exact taxon name in current AMBI list. |
| 87 | Polychaeta | *Scolelepis sp.* | *Scolelepis sp.* | III | Tolerant taxa | Exact | Exact taxon name in current AMBI list. |
| 88 | Polychaeta | *Spio sp.* | *Spio sp.* | III | Tolerant taxa | Exact | Exact taxon name in current AMBI list. |
| 89 | Polychaeta | *Spiophanes sp.* | *Spiophanes sp.* | III | Tolerant taxa | Exact | Exact taxon name in current AMBI list. |
| 90 | Polychaeta | *Sternaspis sp.* | *Sternaspis sp.* | III | Tolerant taxa | Exact | Exact taxon name in current AMBI list. |
| 91 | Polychaeta | *Exogone sp.* | *Exogone sp.* | II | Indifferent taxa | Exact | Exact taxon name in current AMBI list. |
| 92 | Polychaeta | *Typosyllis sp.* | *Typosyllis sp.* | II | Indifferent taxa | Exact | Exact taxon name in current AMBI list. |
| 93 | Polychaeta | *Sphaerosyllis sp.* | *Sphaerosyllis sp.* |  | Unassigned | Unassigned | Taxa unassigned |
| 94 | Polychaeta | *Syllis sp.* | *Syllis sp.* | II | Indifferent taxa | Exact | Exact taxon name in current AMBI list. |
| 95 | Polychaeta | *Terebellidae ñ id.* | Terebellidae | I | Sensitive taxa | Exact | Exact taxon name in current AMBI list. |
| 96 | Polychaeta | *Lysilla sp.* | *Lysilla sp.* | II | Indifferent taxa | Exact | Exact taxon name in current AMBI list. |
| 97 | Polychaeta | *Pista cristata* | *Pista cristata* | I | Sensitive taxa | Exact | Exact taxon name in current AMBI list. |
| 98 | Polychaeta | *Pista corrientis* | *Pista corrientis* | I | Sensitive taxa | Exact | Exact taxon name in current AMBI list. |
| 99 | Polychaeta | *Polycirrus sp.* | *Polycirrus sp.* | IV | Second-order opportunists | Exact | Exact taxon name in current AMBI list. |
| 100 | Polychaeta | *Terebellides anguicomus* | *Terebellides anguicomus* | I | Sensitive taxa | Exact | Exact taxon name in current AMBI list. |
| 101 | Polychaeta | *Trochochaetidae ñ id.* | — |  | Unassigned | Unassigned | Taxa unassigned |
| 102 | Crustacea | Amphipoda | — |  | Unassigned | Unassigned | Taxa unassigned |
| 103 | Crustacea | Ampeliscidae | Ampeliscidae | I | Sensitive taxa | Exact | Exact taxon name in current AMBI list. |
| 104 | Crustacea | *Caprella sp.* | *Caprella sp.* | II | Indifferent taxa | Exact | Exact taxon name in current AMBI list. |
| 105 | Crustacea | Gammaridae | Gammaridae | I | Sensitive taxa | Exact | Exact taxon name in current AMBI list. |
| 106 | Crustacea | Hyalellidae | — |  | Unassigned | Unassigned | Taxa unassigned |
| 107 | Crustacea | *Monocorophium sp.* | *Monocorophium sp.* | III | Tolerant taxa | Exact | Exact taxon name in current AMBI list. |
| 108 | Crustacea | Melphidippidae | — |  | Unassigned | Unassigned | Taxa unassigned |
| 109 | Crustacea | Leucothoidae | — |  | Unassigned | Unassigned | Taxa unassigned |
| 110 | Crustacea | Lysianassidae | Lysianassidae | I | Sensitive taxa | Exact | Exact taxon name in current AMBI list. |
| 111 | Crustacea | Oedicerotidae | Oedicerotidae | I | Sensitive taxa | Exact | Exact taxon name in current AMBI list. |
| 112 | Crustacea | Stilipedidae | — |  | Unassigned | Unassigned | Taxa unassigned |
| 113 | Crustacea | Cumacea | Cumacea | I | Sensitive taxa | Exact | Exact taxon name in current AMBI list. |
| 114 | Crustacea | *Emerita brasiliensis* | — |  | Unassigned | Unassigned | Taxa unassigned |
| 115 | Crustacea | Ocypoddidae | — |  | Unassigned | Unassigned | Taxa unassigned |
| 116 | Crustacea | Penaeidae | Penaeidae | II | Indifferent taxa | Exact | Exact taxon name in current AMBI list. |
| 117 | Crustacea | Mysidacea | — |  | Unassigned | Unassigned | Taxa unassigned |
| 118 | Crustacea | Paguridae | Paguridae | II | Indifferent taxa | Exact | Exact taxon name in current AMBI list. |
| 119 | Crustacea | Isopoda | — |  | Unassigned | Unassigned | Taxa unassigned |
| 120 | Crustacea | *Ancinus sp.* | *Ancinus sp.* | I | Sensitive taxa | Exact | Exact taxon name in current AMBI list. |
| 121 | Crustacea | *Cirolana sp.* | *Cirolana sp.* | II | Indifferent taxa | Exact | Exact taxon name in current AMBI list. |
| 122 | Crustacea | *Pseudosphaeroma sp.* | — |  | Unassigned | Unassigned | Taxa unassigned |
| 123 | Crustacea | *Rocinela signata* | — |  | Unassigned | Unassigned | Taxa unassigned |
| 124 | Crustacea | Ostracoda | Ostracoda |  | Unassigned | Exact | Exact taxon name in current AMBI list. |
| 125 | Crustacea | Pycnogonida | Pycnogonida | II | Indifferent taxa | Exact | Exact taxon name in current AMBI list. |
| 126 | Crustacea | *Monokalliapseudes schubarti* | *Monokalliapseudes schubarti* | II | Indifferent taxa | Exact | Exact taxon name in current AMBI list. |
| 127 | Bryozoa | Bryozoa | Bryozoa | II | Indifferent taxa | Exact | Exact taxon name in current AMBI list. |
| 128 | Echinodermata | Echinoidea | Echinoidea | I | Sensitive taxa | Exact | Exact taxon name in current AMBI list. |
| 129 | Echinodermata | Holothuria | — |  | Unassigned | Unassigned | Taxa unassigned |
| 130 | Echinodermata | Ophiuroidea | Ophiuroidea | II | Indifferent taxa | Exact | Exact taxon name in current AMBI list. |
| 131 | Echinodermata | *Hemipholis elongata* | *Hemipholis elongata* | I | Sensitive taxa | Exact | Exact taxon name in current AMBI list. |
| 132 | Echinodermata | *Ophionereis sp.* | — |  | Unassigned | Unassigned | Taxa unassigned |
| 133 | Echinodermata | *Ophiothela mirabilis* | *Ophiothela mirabilis* | II | Indifferent taxa | Exact | Exact taxon name in current AMBI list. |
| 134 | Bivalvia | *Bivalvia - n ident* | — |  | Unassigned | Unassigned | Taxa unassigned |
| 135 | Bivalvia | *Anomalocardia brasiliana* | *Anomalocardia brasiliana* | I | Sensitive taxa | Exact | Exact taxon name in current AMBI list. |
| 136 | Bivalvia | *Corbula caribaea* | *Corbula caribaea* | IV | Second-order opportunists | Exact | Exact taxon name in current AMBI list. |
| 137 | Bivalvia | *Crassostrea sp.* | — |  | Unassigned | Unassigned | Taxa unassigned |
| 138 | Bivalvia | *Ctena sp.* | *Ctena sp.* | I | Sensitive taxa | Exact | Exact taxon name in current AMBI list. |
| 139 | Bivalvia | *Ervilia sp.* | *Ervilia sp.* | I | Sensitive taxa | Exact | Exact taxon name in current AMBI list. |
| 140 | Bivalvia | *Felaniella vilardeboana* | — |  | Unassigned | Unassigned | Taxa unassigned |
| 141 | Bivalvia | *Lucina pectinata* | *Lucina pectinata* | I | Sensitive taxa | Exact | Exact taxon name in current AMBI list. |
| 142 | Bivalvia | *Macoma sp.* | *Macoma sp.* |  | Unassigned | Unassigned | Taxa unassigned |
| 143 | Bivalvia | *Mactra sp.* | *Mactra sp.* | I | Sensitive taxa | Exact | Exact taxon name in current AMBI list. |
| 144 | Bivalvia | *Mesodesma sp.* | — |  | Unassigned | Unassigned | Taxa unassigned |
| 145 | Bivalvia | *Mytella guyanensis* | *Mytella guyanensis* | I | Sensitive taxa | Exact | Exact taxon name in current AMBI list. |
| 146 | Bivalvia | *Nuculana sp* | — |  | Unassigned | Unassigned | Taxa unassigned |
| 147 | Bivalvia | *Protothaca sp.* | *Protothaca sp.* | II | Indifferent taxa | Exact | Exact taxon name in current AMBI list. |
| 148 | Bivalvia | *Semele sp.* | *Semele sp.* | II | Indifferent taxa | Exact | Exact taxon name in current AMBI list. |
| 149 | Bivalvia | *Sphenia sp.* | *Sphenia sp.* | I | Sensitive taxa | Exact | Exact taxon name in current AMBI list. |
| 150 | Bivalvia | *Tellina sp.* | *Tellina sp.* | I | Sensitive taxa | Exact | Exact taxon name in current AMBI list. |
| 151 | Bivalvia | *Tagelus divisus* | *Tagelus divisus* | II | Indifferent taxa | Exact | Exact taxon name in current AMBI list. |
| 152 | Gastropoda | *Gastropoda - n ident* | — |  | Unassigned | Unassigned | Taxa unassigned |
| 153 | Gastropoda | *Assiminea succinea* | *Assiminea succinea* | II | Indifferent taxa | Exact | Exact taxon name in current AMBI list. |
| 154 | Gastropoda | *Bulla striata* | *Bulla striata* | II | Indifferent taxa | Exact | Exact taxon name in current AMBI list. |
| 155 | Gastropoda | *Calyptraea centralis* | — |  | Unassigned | Unassigned | Taxa unassigned |
| 156 | Gastropoda | *Cosmioconcha helenae* | — |  | Unassigned | Unassigned | Taxa unassigned |
| 157 | Gastropoda | *Cyclostremiscus beauii* | — |  | Unassigned | Unassigned | Taxa unassigned |
| 158 | Gastropoda | *Crepidula sp* | *Crepidula sp.* | III | Tolerant taxa | Exact | Exact taxon name in current AMBI list. |
| 159 | Gastropoda | *Heleobia australis* | *Heleobia australis* | IV | Second-order opportunists | Exact | Exact taxon name in current AMBI list. |
| 160 | Gastropoda | *Leuocozonia nassa* | — |  | Unassigned | Unassigned | Taxa unassigned |
| 161 | Gastropoda | *Natica sp.* | *Natica sp.* | II | Indifferent taxa | Exact | Exact taxon name in current AMBI list. |
| 162 | Gastropoda | *Neritina virginea* | *Neritina virginea* | I | Sensitive taxa | Exact | Exact taxon name in current AMBI list. |
| 163 | Gastropoda | *Odostomia sp.* | *Odostomia sp.* | II | Indifferent taxa | Exact | Exact taxon name in current AMBI list. |
| 164 | Cnidaria | Anthozoa | Anthozoa | II | Indifferent taxa | Exact | Exact taxon name in current AMBI list. |
| 165 | Cnidaria | *Renilla sp.* | — |  | Unassigned | Unassigned | Taxa unassigned |
| 166 | Nemertea | Nemertea | Nemertea | III | Tolerant taxa | Exact | Exact taxon name in current AMBI list. |
| 167 | Phoronida | Phoronida | Phoronida | II | Indifferent taxa | Exact | Exact taxon name in current AMBI list. |
| 168 | Sipuncula | Sipuncula | Sipuncula | I | Sensitive taxa | Exact | Exact taxon name in current AMBI list. |
| 169 | Cepahlochordata | *Branchiostoma sp.* | *Branchiostoma sp.* | I | Sensitive taxa | Exact | Exact taxon name in current AMBI list. |

**Supplementary Table S2.** AMBI and M-AMBI values for each station sampled in this study, with ecological variables used in M-AMBI calculations.S= species richness, H’= Shannon diversity

| **Estuary/Station** | **AMBI** | **SD** | **Ecological group (% relative abundance)** | | | | | **Abundance** | **S** | **H'** | **M-AMBI** | **Classification** |
| --- | --- | --- | --- | --- | --- | --- | --- | --- | --- | --- | --- | --- |
| **Vitoria Bay** |  |  | I | II | III | IV | V |  |  |  |  |  |
| BV 02 | 3.5 | 0.2 | 5.9 | 24 | 24.5 | 25.1 | 20.5 | 900 | 32 | 3.86 | 0.60 | Moderate |
| BV 04 | 2.5 | 0.2 | 29.5 | 22.1 | 12.1 | 26.8 | 9.6 | 1093 | 51 | 4.35 | 0.77 | Good |
| BV 07 | 3.7 | 0.2 | 11 | 12.2 | 19.5 | 36.3 | 20.9 | 1151 | 23 | 3.33 | 0.50 | Moderate |
| BV 09 | 2.2 | 0.1 | 13.9 | 51.1 | 15.9 | 15.6 | 3.5 | 749 | 47 | 4.2 | 0.74 | Good |
| BV 12 | 3.3 | 0.1 | 13.4 | 14.4 | 28.7 | 28 | 15.4 | 2596 | 75 | 4.98 | 0.88 | Good |
| BV 15 | 2.4 | 0.1 | 22.1 | 28.7 | 23 | 16.4 | 9.8 | 153 | 41 | 4.5 | 0.74 | Good |
| BV 17 | 5.1 | 0.4 | 5.5 | 7.6 | 4.7 | 1.9 | 80.3 | 941 | 27 | 1.75 | 0.31 | Poor |
| BV 19 | 2.0 | 1.0 | 32.7 | 30.3 | 9 | 25.5 | 2.5 | 668 | 45 | 4.06 | 0.73 | Good |
| BV 21 | 2.3 | 0.2 | 17.6 | 22.4 | 53.5 | 1.8 | 4.7 | 181 | 26 | 3.69 | 0.62 | Good |
| BV 24 | 1.5 | 0.0 | 17.9 | 71.4 | 0 | 10.7 | 0 | 31 | 13 | 3.28 | 0.55 | Good |
| BV 27 | 2.2 | 0.5 | 6 | 38.6 | 55.4 | 0 | 0 | 90 | 20 | 3.41 | 0.57 | Good |
| BV 30 | 1.8 | 0.4 | 36.8 | 3.8 | 58.5 | 0.9 | 0 | 267 | 17 | 2.29 | 0.48 | Good |
| BV 32 | 1.3 | 0.7 | 59.7 | 17.9 | 16.4 | 5.2 | 0.7 | 146 | 31 | 3.52 | 0.66 | Good |
| BV 34 | 1.7 | 0.1 | 20.5 | 56.4 | 12.8 | 10.3 | 0 | 42 | 17 | 3.73 | 0.60 | Good |
| BV 36 | 1.0 | 0.2 | 31.5 | 64.6 | 1.7 | 2.2 | 0 | 301 | 21 | 3.59 | 0.64 | High |
| CP 02 | 3.1 | 1.1 | 26.9 | 17.5 | 9.4 | 24.5 | 21.7 | 263 | 27 | 4.07 | 0.60 | Good |
| CP 04 | 6.0 | 1.5 | 0 | 13.3 | 6.7 | 20 | 60 | 15 | 6 | 2.29 | 0.23 | Bad |
| CP 06 | 2.5 | 0.8 | 28.2 | 22.1 | 22.9 | 19.1 | 7.6 | 134 | 19 | 3.67 | 0.57 | Good |
| CP 07 | 4.1 | 1.1 | 15.7 | 18.8 | 8.2 | 11.8 | 45.5 | 665 | 28 | 3.02 | 0.48 | Moderate |
| **Piraquê-Açú-Mirim** |  |  |  | | | | |  |  |  |  |  |
| PA01 | 1.8 | 0.0 | 6.5 | 67.4 | 26.1 | 0 | 0 | 57 | 2.1956 | 6 | 0.51 | Good |
| PA03 | 0.9 | 0.3 | 62.8 | 22.5 | 11.3 | 0 | 3.5 | 244 | 2.329 | 16 | 0.64 | High |
| PA05 | 1.8 | 0.1 | 34 | 34.9 | 8 | 23 | 0 | 710 | 3.7063 | 39 | 0.82 | Good |
| PA07 | 2.2 | 0.7 | 21.6 | 29.9 | 16.5 | 31.6 | 0.3 | 322 | 4.0885 | 34 | 0.80 | Good |
| PA08 | 1.9 | 0.2 | 25.5 | 43.3 | 9.8 | 21.2 | 0.2 | 1167 | 4.2721 | 49 | 0.92 | Good |
| PA09 | 1.6 | 0.0 | 49.4 | 20.2 | 6.7 | 23.6 | 0 | 380 | 4.2318 | 34 | 0.85 | Good |
| PA10 | 1.0 | 0.1 | 58.2 | 25.6 | 7.5 | 5.5 | 3.2 | 374 | 3.1802 | 30 | 0.78 | High |
| PA11 | 1.4 | 0.2 | 13.6 | 79.4 | 4.4 | 2.5 | 0 | 349 | 3.3539 | 35 | 0.80 | Good |
| PM01 | 1.7 | 0.1 | 21.5 | 49.7 | 20.1 | 8.8 | 0 | 723 | 4.3601 | 43 | 0.90 | Good |
| PM05 | 1.6 | 0.1 | 36.2 | 44.8 | 8.6 | 0 | 10.3 | 65 | 2.9601 | 15 | 0.64 | Good |
| PM06 | 1.2 | 0.2 | 31.9 | 59.6 | 6.4 | 0 | 2.1 | 52 | 1.5904 | 6 | 0.50 | High |

**Supplementary Figures**

**Supplementary Figure S1.** Macrofaunal abundance and diversity across estuarine sectors. Violin plots show abundance (individuals m⁻²), taxonomic richness (S), Shannon diversity (H′, log₂) and Pielou evenness (J′) calculated separately for each 0.1-m² Day-grab sample. Internal boxplots represent the median and interquartile range, and points represent individual grab samples. For station-level community analyses, taxon counts from the two grabs were summed by taxon and richness, Shannon diversity and evenness were recalculated from the pooled station assemblage; biodiversity metrics were not added or averaged across grabs. Vitória Bay (VB; shown as BV in the current rendered panel) includes Canal da Passagem (CP) stations.

**Supplementary Figure S2.** Macrofaunal composition, dominance and station-level diversity across the study area. The upper matrix shows the 20 most abundant taxa; tile colour represents log₁₀(N + 1) abundance and white circles show within-station relative abundance. The three lower rows show station-level taxonomic richness (S), Shannon diversity (H′, log₂) and Pielou evenness (J′). Numbers are raw station-level values; the separate viridis ramp indicates each station’s relative position from low to high within each metric. Station-level diversity metrics were recalculated after summing the two Day-grab taxon counts by station. Vitória Bay (VB) includes Canal da Passagem (CP) stations.

**Supplementary Figure S3.** Station-resolved comparison of M-AMBI ecological quality and AMBI disturbance. Panel (a) shows M-AMBI values and ecological-status classes using a viridis ecological-quality palette; panel (b) shows AMBI values and disturbance conditions using the reversed magma palette. Fixed-size diamonds show station values, black long-dashed lines show the overall means (M-AMBI = 0.61; AMBI = 2.23), and grey dotted lines in the AMBI panel show the numeric disturbance boundaries. Stations are ordered by decreasing M-AMBI within each sector. Vitória Bay (VB; shown as BV in the current rendered panel) includes Canal da Passagem (CP) stations.

**Supplementary Figure S4.** Station-to-sector pathways across AMBI disturbance conditions. Each alluvial ribbon represents one sampling station. The left axis identifies stations, the middle axis separates Vitória Bay, Piraquê-Açu and Piraquê-Mirim, and the right axis shows the AMBI disturbance condition. Ribbon colours identify estuarine sectors, whereas disturbance-condition strata use the coordinated reversed-magma AMBI palette. Vitória Bay includes both VB and Canal da Passagem (CP) stations.

**Supplementary Figure S5.** Descriptive environmental summary for the matched 30-station subset. Panel (a) shows log(X + 1)-transformed environmental variables standardized within predictors (z-scores), with stations grouped by sector. Panel (b) shows an unconstrained PCA after variable-wise Yeo-Johnson transformation and z-standardization; dashed curves are 80% descriptive data ellipses for Vitória Bay + Canal da Passagem (VB + CP) and Piraquê-Açu + Piraquê-Mirim (PA + PM), and arrows show the strongest displayed environmental loadings. The PCA is descriptive and was not used for hypothesis testing, predictor selection or inference about macrofaunal responses. Pb is excluded because the PA10 value was physically invalid and no laboratory-verified replacement was available.

**Supplementary Figure S6.** Study-area maps showing the full station network, the subset of 30 stations used for matched geochemical analyses, major urban/industrial areas, and mangrove distribution. Panel (a) shows the Piraquê-Açu–Piraquê-Mirim estuarine system and panel (b) shows Vitória Bay. Circle fill indicates taxonomic richness using the same low-to-high richness scale as the study-area richness map. Stations included in the matched geochemical subset are emphasized with an additional black outer ring. Urban/industrial areas are shown as light shaded polygons and mangrove areas are displayed with a contrasting non-green fill to avoid overlap with the richness colour ramp. Map component sources: GEOIEMA and ggplot2 package in R.
